# Conditions near a crack tip: Advanced experiments for dislocation analysis and local strain measurement

**DOI:** 10.1557/s43577-022-00377-4

**Published:** 2022-08-24

**Authors:** Christoph Gammer, Dayong An

**Affiliations:** 1 Erich Schmid Institute of Materials Science, Austrian Academy of Sciences, Leoben, Austria; 2 Department of Plasticity Technology, School of Materials Science and Engineering, Shanghai Jiao Tong University, Shanghai, China

**Keywords:** Fracture, Strain, 4D-STEM, Electron backscatter diffraction (EBSD), X-ray diffraction

## Abstract

The local stress state and microstructure near the crack-tip singularity control the fracture process. In ductile materials multiple toughening mechanisms are at play that dynamically influence stress and microstructure at the crack tip. In metals, crack-tip shielding is typically associated with the emission of dislocations. Therefore, to understand crack propagation on the most fundamental level, *in situ* techniques are required that are capable to combine imaging and stress mapping at high resolution. Recent experimental advances in x-ray diffraction, scanning electron microscopy, and transmission electron microscopy enable quantifying deformation stress fields from the bulk level down to the individual dislocation. Furthermore, through modern detector technology the temporal resolution has sufficiently improved to enable stress mapping during *in situ* experiments.

**Graphical abstract:**

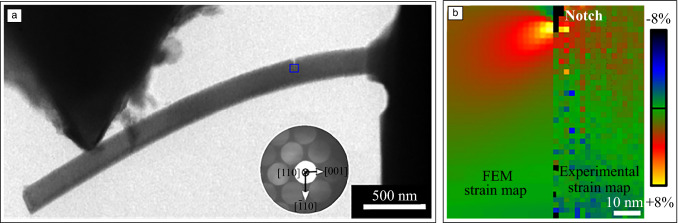

## Introduction

Understanding failure of materials under an applied load is of central importance for their application. Therefore, fracture mechanics has become a well-established field, trying to predict the conditions necessary for crack growth. Whereas various parameters have been successfully used to quantify crack-tip driving forces, in materials that are not ideally brittle, fracture mechanisms are typically very complex due to various crack-tip shielding mechanisms being at play.^1^ The situation is even more complicated in heterogeneous or multiphase materials. As the structure at the crack tip is dynamic the local conditions need to be measured *in situ* for obtaining a full understanding of the fundamental fracture processes at play. Importantly, crack propagation, defect evolution, and local stress changes need to be imaged at the same time. Therefore, advanced experimental techniques are required that not only offer sufficient resolution to image the crack tip, but also allow quantifying local strains or microstructural features such as dislocation densities around the crack tip. Recently, there have been enormous advances in both imaging and diffraction-based techniques for quantifying strain states or deformation microstructures in materials. In this article, the most important techniques for imaging the crack tip such as x-ray tomographic imaging or electron microscopy-based techniques will be reviewed.

To quantify the deformation around a crack tip, digital image correlation (DIC) is routinely used allowing to measure the total strain by tracking features on the specimen.^2^ More important for predicting the crack propagation is the elastic strain in front of the crack tip. Knowing the elastic component of the total strain, the plastic strain can be deduced. The local elastic strain is directly related to the local stress,^3^ which is typically measured through local diffraction techniques allowing to determine changes in lattice parameters. Depending on the specific application, neutron diffraction,^4^ x-ray diffraction (XRD),^5^ or electron diffraction^6^ is used. Finally, recent advances of electron backscatter diffraction (EBSD) in the scanning electron microscope (SEM) have demonstrated the possibility to map strain at high resolution during deformation.^7^ Although the developments have brought these techniques closer together in terms of their spatial resolution, it is still important to take into account the sample size and scale of the microstructural features of interest. While TEM offers sufficient resolution to map strain in nano-volumes, XRD allows mapping bulk specimens in a nondestructive manner. Finally, SEM allows imaging at high resolution, but is traditionally surface-sensitive. In the following chapters XRD, SEM, and TEM techniques are reviewed in light of their specific advantages for studying the conditions at the crack tip.

### X-ray diffraction

**Figure 1:**
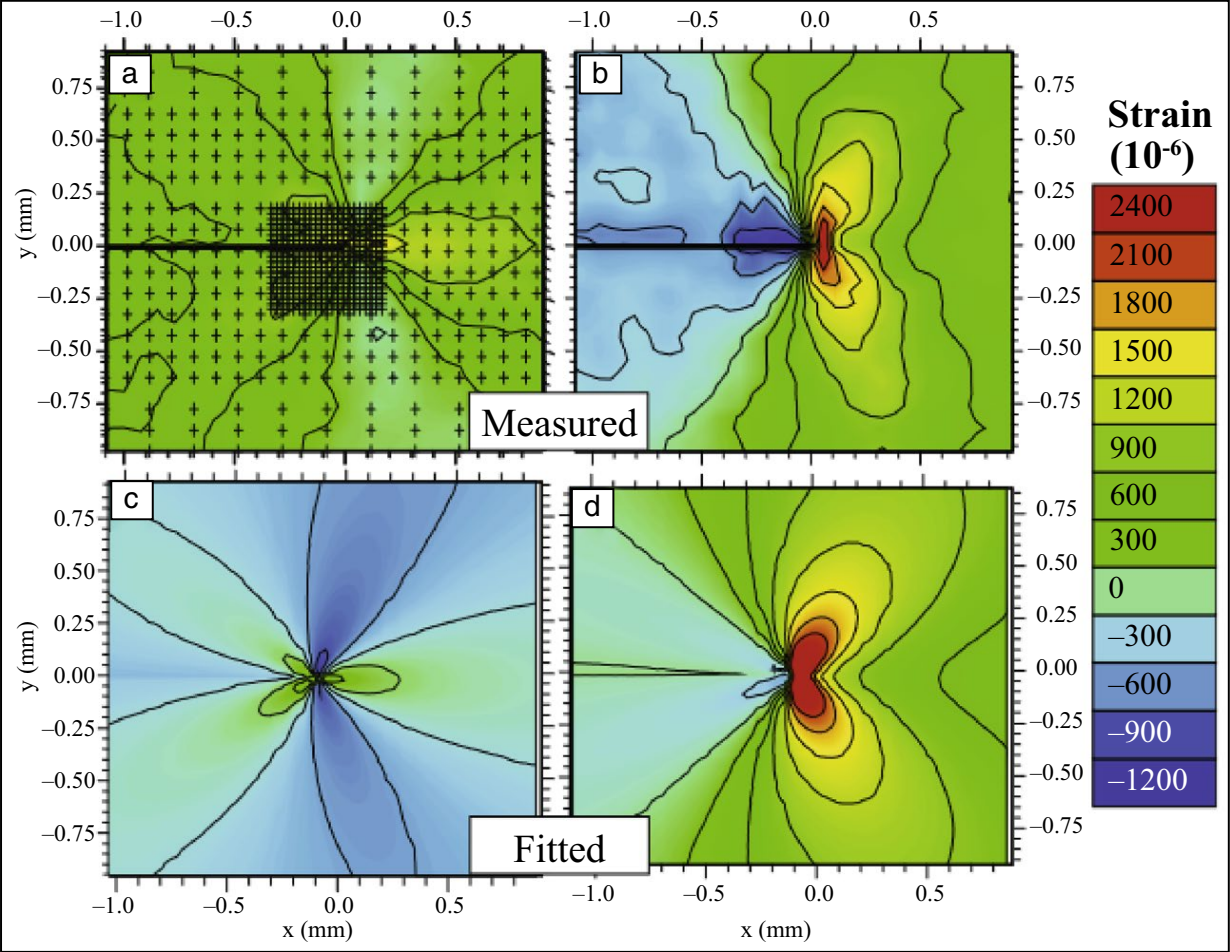
Strain mapping around a crack tip in an ultrafine-grained aluminum alloy using x-ray diffraction. The raw data (a, b) and best fit elastic strain fields (c, d) are shown for the crack growth direction (left) and crack opening direction (right).^11^

XRD is a well-established technique for measuring various microstructural properties of materials. Of central importance is the possibility to measure reciprocal lattice vectors with enough precision to accurately determine strain. By using multiple reflections, a strain tensor can be obtained, which is typically converted to a stress using the elasticity constants of the materials.^8^ XRD has been used for decades measuring the stress in the vicinity of cracks. Recent advances in synchrotron sources allow crack-tip strain fields to be mapped deep within a material using hard x-rays.^9^ This is a fundamental advantage of XRD, as it allows to reveal the conditions near a crack tip in bulk specimens. While traditionally XRD yields one global stress value, recently the use of finely focused synchrotron radiation has enabled to scan the beam over the specimen, allowing to determine strain locally. Nowadays, various beamlines offer a resolution below 100 nm and fast detectors enable to record a 2D map containing a full diffraction pattern for each probe position. Therefore, local strain maps around a crack tip can be measured during loading.^10^ Apart from instrumental limitations, an inherent limitation of diffraction-based strain measurements is based on the grain size in relation to the scattering volume. In the case of polycrystalline materials, powder diffraction techniques need to be used that require sufficient grains to be illumined by the x-ray beam. Therefore, this technique is typically applied to ultrafine-grained or nanocrystalline materials.^10^ **Figure ****1** shows the measured strain field around a crack in an ultrafine-grained aluminum alloy, along with the best fit theoretical crack-tip strain field based on linear elastic fracture mechanics.^11^ From the direct comparison, the crack-tip stress intensity factor can be deduced. In addition to the evaluation of the peak position, peak broadening can be measured in XRD, if crystallite sizes are sufficiently small or if lattice defects are present. Although a direct quantification of dislocation densities can be challenging, the peak broadening is a good indicator for defects, allowing to estimate the plastic zone size. Traditionally, depth sectioning was used to measure the crack-tip plastic zone size postmortem; however, mapping in the synchrotron now enables the visualization of the plastic zone size along with the local strain field around a crack tip.^12^ Of equal importance is the possibility to directly image the crack, allowing the measurement of the crack-tip opening or deducing the crack path, which can be nowadays correlated with the local stress state of individual grains.^13^ Specifically, x-ray tomography has been shown to be a valuable tool, both for postmortem studies, or for interrupted imaging during fatigue testing. A specific benefit of XRD is the possibility to use a variety of deformation stages and sample geometries for *in situ* deformation, allowing to investigate fracture in real time under realistic conditions.^14^ The possibility to monitor cracks deep within and linking the crack tip to microstructural features with increasing resolution enables monitoring the conditions at the crack tip in a growing range of materials.

### Scanning electron microscopy

#### Strain and dislocation analysis by high angular- and spatial-resolution EBSD

EBSD and EBSD-based orientation microscopy allow one to quantitatively obtain information about crystallographic orientation, phase composition, and defect densities based on the analysis of EBSD patterns.^15^ By using the Hough transformation, the nearly straight Kikuchi bands in the EBSD patterns are converted to points, which can be automatically located by the software.^16^ With increasing automation, EBSD became one of the most frequently used techniques for materials scientists and geologists.^17^ An important application of EBSD is strain analysis.^7^ Both elastic and plastic strain can affect the EBSD pattern. Quantitative analyses of elastic and plastic strain are however still challenging, due to the limitation of angular and spatial resolution of EBSD measurements.^18^ Nevertheless, significant progress has been made for accurate analysis of strains and dislocations, such as cross-correlation methods^19,20^ and transmission Kikuchi diffraction (t-EBSD or TKD),^21,22^ which will be highlighted in the following section.

The angular resolution of Hough transformation-based EBSD is around 0.5–2°.^23^ An accuracy of 0.1–0.2° was achieved by developing new techniques, namely the dictionary^24^ and the spherical harmonic^25^ transform-based approaches. Using the cross-correlation-based EBSD (CC-EBSD) method introduced by Wilkinson et al.,^19^ the sensitivity for elastic strain and lattice curvature measurement has improved to 0.005°. Cross-correlation is used to determine small shifts in the patterns compared to a reference pattern for a number of regions of interest dispersed across the obtained EBSD patterns. If more than four regions of interest are selected, the displacement gradient tensor can be solved for the best solution in a least-square sense. The symmetric and antisymmetric components of the displacement gradient tensor yield the elastic strains and lattice rotations. Strain is given relative to the reference, whereas the strain state of the reference point is typically unknown. This remains the most significant challenge for measuring absolute strains.

Geometrically necessary dislocation (GND) density can be calculated according to Nye’s theory,^26^ from the lattice rotation tensor. The theory was further extended by Kröner,^27^ considering the elastic strain tensor. Note that GND measurements are mostly based on two-dimensional EBSD (2D-EBSD) results, which lead to the lack of out-of-plane components in Nye’s dislocations. However, Pantleon^28^ showed that a remarkable improvement can be achieved by considering all available components of the lattice curvature tensor. To exclude effects from the incomplete Nye tensor, Konijnenberg et al.^29^ verified the density of GNDs in a copper bicrystal under a well-controlled microcantilever bending experiment by 3D-EBSD. The GND density determined experimentally was in good accordance with the theoretical assessment.

**Figure 2:**
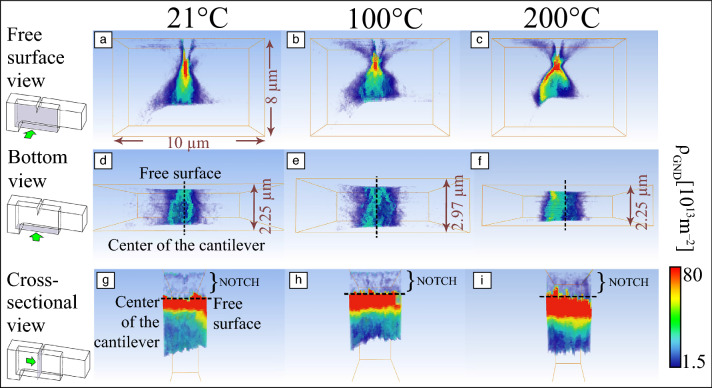
Total geometrically necessary dislocation density reconstructions calculated using 3D cross-correlation-based-electron backscatter diffraction.^31^ Tungsten single-crystal cantilevers deformed at three different temperatures (21°C, 100°C, and 200°C with 5 nm/s tip velocity), viewed from the free surface (a–c), from the bottom (d–f), and the cross section (g–i) of the sample at the crack position. Dashed lines indicate the position of the crack front. Three-dimensional mapping was carried out only on the half volume of each cantilever. Sketches on the side in each row show with gray areas from where the reconstruction is viewed.

Wilkinson et al.^20^ reported that in many cases the lattice rotation gradients are considerably larger (normally more than an order of magnitude) than the elastic strain gradients, and it is thus reasonable to neglect the elastic parts or include only the available terms. Note that there are more GND types than the number of available strain components, and therefore the solution of GND types is not unique. In CrossCourt,^7^ which is a commercial software for CC-EBSD, GNDs are calculated by seeking a combination of GND types that fit all available components and have the minimum total line energy.^30^ CC-EBSD has proved to be a powerful technique to investigate the crack propagation process. For example, Kalácska et al.^31^ investigated the shape of the plastic zone around crack tips in a W single crystal in terms of GNDs using CC-EBSD combined with focused ion beam (FIB) in three dimensions (i.e., 3D CC-EBSD), as shown in **Figure ****2**. Cantilevers with a notch were deformed inside the SEM at different temperatures. It can be seen that the GND densities near the free surface were found to be higher than those inside the specimen at all three temperatures. However, the 3D shape of the plastic zone changes from being localized in front of the crack tip to butterfly-like distributions at higher temperature, which shields more efficiently the crack tip and consequently inhibits crack propagation. The underlying mechanism is ascribed to the more intensely activated screw GNDs at higher deformation temperature.

Apart from angular resolution, the spatial resolution of EBSD also plays an important role for strain and dislocation analysis (e.g., determination of GND density).^32^ Despite the high angular resolution achieved by cross-correlation-based methods, the spatial resolution remains unchanged. The TKD technique introduced by Keller and Geiss,^21^ where thin foils (50–150 nm) are observed in transmission mode in the SEM, significantly improves the spatial resolution down to 10 nm. By combining TKD with CC-EBSD, namely HR-TKD, a combination of high angular and spatial resolution can be achieved. Yu et al.^33^ demonstrated that the details of lattice distortions near an individual dislocation can be resolved by HR-TKD. Fundenberger et al.^34^ introduced an “on-axis” configuration, which enables faster acquisition time, lower pattern distortion, and a slightly better spatial resolution compared to the conventional “off-axis” configuration. By coupling the cross-correlation algorithms and on-axis TKD, Ernould et al.^35^ could investigate the grain internal disorientations and GND densities of a nanocrystalline pure aluminum. Wang et al.^36^ reported that by applying a monolithic active pixel sensor-based direct electron detector, high-quality EBSD patterns or high-speed pattern acquisition (up to 5988 fps) can be achieved. The high temporal resolution can greatly facilitate the dynamic *in situ* deformation experiments.

#### Dislocation analysis by electron channeling contrast imaging (ECCI)

Electron channeling contrast imaging (ECCI) can be used for direct observation of crystal lattice defects in bulk samples (e.g., dislocations, stacking faults, and grain boundaries), which are commonly investigated using TEM of thin foils. To obtain optimum contrast for ECCI observation, the two-beam diffraction condition is required, where only one set of lattice planes is tilted into Bragg condition. In general, there are two types of approaches to establish channeling conditions (i.e., by collecting a selected area channeling pattern^37^ or by a simulation of electron channeling patterns [ECPs]), based on EBSD measurements.^30,38^ In the following, recent progress of these two methods will be highlighted.

ECPs, which are Kikuchi-like patterns similar to the EBSD patterns, were first observed by Coates^39^ on a topographical image obtained with BSD. ECPs can be obtained at low magnifications, where the orientation relationship between incident beam and lattice plane varies over the field of view. When the incident beam satisfies the Bragg condition for a particular set of lattice planes, the perfect crystal is illuminated in channeling conditions (i.e., the backscattering electron intensity is weak). Alternatively, ECPs can also be produced by rocking the beam over a fixed point on the sample and recording the backscattering electron intensities over different beam incident angles.^37^ By collecting high-resolution selected area channeling patterns overlaid with the simulated EBSD patterns, Mansour et al.^40^ developed an accurate ECCI (A-ECCI) approach to control the channeling condition with sub-micron spatial resolution (approaching around 125 nm as reported in a recent study^41^) and high angular accuracy of about 0.1°. A-ECCI allows a broad range of applications for crystal defects characterization, such as the determination of the crystallographic information of dislocations^42^ and low angle subgrain boundaries.^40^

Another approach to control the channeling condition for performing ECCI is based on simulated ECP using, for example, the software “Tools for orientation determination and crystallographic analysis” (TOCA),^43^ which is known as ECCI under controlled diffraction conditions (cECCI). Before carrying out ECCI at low tilts, the sample is positioned at a high tilt angle (like 70°) for the EBSD scan of the region of interests. Thereafter, the orientation information based on the EBSD measurement is entered into the TOCA software for ECP simulation. With the assistance of TOCA, the stage tilts and rotations needed to bring a crystal into optimum channeling condition are obtained. It should be mentioned that, due to the accuracy limitations of EBSD and the uncertainty of the SEM stage control, a discrepancy between the TOCA predicted orientation and the real channeling orientation could exist. To decrease this error, a large and reasonably perfect single crystal can be used to measure and calibrate this misalignment. A detailed review on cECCI can be found in Zaefferer et al.^38^

#### Combining ECCI and HR-EBSD for dislocation and strain analysis

**Figure 3:**
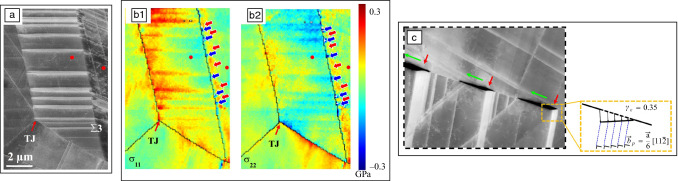
Combining controlled electronic channeling contrast imaging (cECCI) and cross-correlation-electron backscatter diffraction (CC-EBSD) to investigate hydrogen embrittlement of a twinning-induced plasticity steel,^45^ (a) ECCI, (b1, b2) normal stress distribution of hydrogen-charged sample. (c) ECCI showing intergranular crack propagation in the hydrogen-charged sample. The bright lath plates in (a) and (c) are ε-martensites.

SEM-based techniques show great potential for characterization of deformation microstructures. As demonstrated in a number of studies, ECCI and HR-EBSD can complement each other for a better analysis of deformation processes.^30,44,45^ **Figure ****3** shows the benefit of combining cECCI with CC-EBSD for investigating hydrogen embrittlement of a TWIP steel.^45^ The fatigue structure is revealed by ECCI in Figure 3a. Hydrogen was found to assist the formation of ε-martensite in the studied material. The impingement of ε-martensite plates on grain boundaries leads to high local stress concentrations, as revealed by CC-EBSD in Figure 3b1–b2. Furthermore, the stress fields are found to change across the impingement from tensile to compressive values. These stress fields play a dominant role on the observed intergranular crack propagation (i.e., cracks nucleate and propagate along the sites with tensile stress fields while being arrested by the compressive stress field). Besides, the fact that the crack opening angle corresponds almost exactly to the shear strain of the deformation martensite (Figure 3c) suggests that completely brittle intergranular cracking occurs due to a strong hydrogen enhanced decohesion mechanism.

### Transmission electron microscopy

Due to its high-spatial resolution TEM is traditionally used to study deformation-induced changes. Important microstructural features, such as dislocation, grain boundaries, precipitates or voids can be directly imaged in the TEM. A major drawback is however the need to use ultrathin specimens. Modern preparation techniques, such as FIB milling, allow the investigation of specifically chosen regions in a deformed specimen; however, the observable sample volume remains limited.^46^ Still, due to the possibility to directly image defects, TEM remains however essential for studying deformation. While traditionally, specifically chosen imaging conditions, such as weak beam dark-field,^47^ are used for imaging dislocations, recently diffraction contrast scanning TEM (STEM) is increasingly used for imaging dislocations.^48^ The reason is that in STEM, dynamical effects are reduced and dislocations can be imaged despite mistilts in the specimen, as are frequently present in deformed materials. Modern TEMs combine a variety of analytical modes with imaging and diffraction. Therefore, dislocation structures, precipitates, and chemical changes around the crack tip can be measured in the same specimen at high resolution.^49,50^

*In situ* deformation in the TEM is of particular importance for studying deformation in materials due to the unique possibility to directly image dynamic dislocation mechanisms under an external applied stress.^51^ Traditionally, the TEM sample is only locally thinned and deformed in a tensile holder. Stress concentrations in the thin areas of the specimen lead to localized deformation allowing the imaging of important deformation mechanisms such as the movement of individual dislocations and their interaction with obstacles.^52,53^ Tensile holders are widely used and have the advantage that they allow to deform different sample geometries. The use of relatively large specimens that are only locally thinned enables imaging deformation for an extended time before catastrophic fracture occurs.^54^ Alternatively, the use of *in situ* deformation based on MEMS devices or advanced nanoindenter holders offers the ability to quantify the load and displacement during deformation.^55^ In combination with well-defined sample geometries this enables to record a stress–strain curve that can be directly linked with the video recorded during the *in situ* deformation. Specifically, the use of nanoindenter holders allows to perform nanoindentation^56^ or compression tests of nanopillars, deform bending beams,^57^ and finally carry out tensile tests through the use of special grippers or a push-to-pull device.^58^

**Figure 4:**
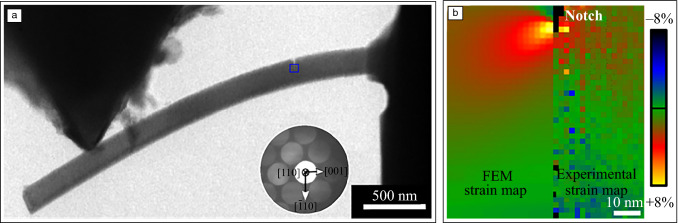
Nanoscale *in situ* transmission electron microscopy fracture experiment on single-crystalline Si.^57^ (a) Scanning transmission electron microscope image showing a 136-nm-thick bending beam under an applied load of 4.1 µN. (b) An experimental strain map in the crack opening direction recorded under load is compared to the strain distribution obtained for the same setup using finite element modeling (FEM).

Recently, the use of *in situ* deformation for studying fracture processes and crack propagation has become increasingly popular. The reasons are the unique possibility to image fundamental deformation processes, the increasing interest in nanostructured materials, and finally the ability to study fracture in small-scale materials.^52,57,59^ Observation of crack tips in metallic specimens during tensile deformation provides direct insight into dislocation emission mechanisms.^60^ This not only allows the investigation of the type and spatial configuration of dislocations that are emitted from the crack tip, but through their curvature even local friction stresses can be estimated.^61^ Thin-foil effects leading to local thinning and dislocation escape have to be taken into account when analyzing the crack propagation.^62^ Therefore, *in situ* deformation is frequently used to follow the crack propagation in nanocrystalline thin films, where the possibility to dynamically image grain orientations and dislocation structures ahead of the crack tip has provided useful information on the governing crack growth processes.^63^ More recently, the fracture behavior of specimens containing nanoscale heterogeneities has attracted increasing interest. Here the crack propagation can be directly linked to the microstructure ahead of the crack tip.^59,64^ Specifically, crack deflection, crack bridging, crack-tip bunting as well as nanocrack formation can be directly imaged. With the combination of tailored fracture sample geometries and modern deformation holders important fracture parameters can be quantified. The possibility to select the region of interest for the crack to initiate allows to determine fracture toughness in individual phases or quantify the effect of individual grain boundaries.^65,66^ Finally, the fact that during *in situ* deformation in the TEM small specimens are used allows to directly evaluate size effects in materials. **Figure ****4**a shows a nanoscale fracture experiment of a single-crystalline Si bending beam. Through a systematic study of different thicknesses, size effects in fracture toughness could be revealed.^57^

While the combination of modern deformation holders with well-defined deformation geometries allows for accurately measuring mean stresses, local stress levels are highly inhomogeneous, requiring the possibility to measure the transient elastic strain field *in situ* during deformation. Specifically, in the vicinity of the crack tip, the strain field shows large gradients. While in the elastic regime and in case of homogeneous materials this can be analytically calculated, that is no longer true after plastic deformation, requiring a direct experimental measurement. In comparison to the XRD- and SEM-based techniques described in the previous chapters, TEM allows measuring strain in nano-volumes.^67^ Strain mapping in the TEM is typically applied to a single well-oriented grain. The most direct method for obtaining strain information is based on the imaging of atomic columns. High-resolution TEM (HRTEM) is frequently used to image the projected lattice and the use of aberration-corrected HRTEM has greatly improved the interpretability. Still, the results strongly depend on the use of thin perfectly oriented specimens. Whereas this is difficult to achieve in traditional *in situ* experiments, where local mistilts during deformation are unavoidable, it has been successfully employed to image fracture in ultrathin metallic films^68^ or two-dimensional materials.^64^ As an alternative, high-angle annular dark-field (HAADF) STEM can be used for directly imaging the atomic lattice. Both drift and scanning artefacts will, however, strongly influence the results, making the applicability to dynamic *in situ* deformation experiments challenging. Therefore, scanning nanobeam electron diffraction (NBED) is becoming increasingly popular. This technique is based on 4D-STEM, where during STEM imaging, a full diffraction pattern is recorded for each probe position.^69^ Conventional NBED uses a rather parallel beam, but recently, a more converged electron beam is employed, allowing to reach a subnanometer resolution.^70^ The sample has to be tilted to zone-axis and the strain mapping is based on determining the changes in the positions of the diffracted peaks. In contrast to standard STEM, where a monolithic detector is used to acquire a single intensity value for each probe position, in 4D-STEM the entire diffraction pattern has to be acquired, requiring long exposure time. Therefore, experiments combining 4D-STEM with *in situ* deformation have tremendously benefited from the advent of ultrafast direct electron detectors.^70^ The possibility of measuring the transient elastic strain at the nanoscale can be very valuable for analyzing the crack tip. Therefore, it is expected that it will find increasing application in this field. This is demonstrated for the silicon bending beam in Figure 4b, where the strain map recorded around the notch tip during elastic loading shows good agreement with the finite element modeling.

## Conclusions

Owing to novel instrumentation and more advanced experimental techniques, our capabilities to image defects and measure local strain fields in highly deformed materials have tremendously improved. Currently, materials science has a large toolset at hand that ranges from revealing buried cracks in bulk materials to imaging single dislocations at high resolution. Correlative techniques and targeted sample preparation allow to analyze materials in-depth after failure. However, to fully understand the dynamical nature of the crack tip, *in situ* deformation experiments are required. Recently, there have been significant advances in both, deformation stages and sample geometries, allowing more controlled *in situ* fracture experiments. Increasingly, these investigations allow to extract quantitative parameters. Still, to fully understand fracture in ductile materials, the strain field around the crack tip is required. While DIC yields the total strain during deformation, only through an accurate measurement of the local elastic strain the individual strain or stress components at the crack tip can be obtained. Local elastic strain mapping is now routinely achievable using XRD, SEM, and TEM. All of these techniques have their strength and weaknesses. Therefore, the optimum sample size and required resolution have to be carefully chosen before planning an experiment in light of, for example, grain or plastic zone size. While to date generally individual experiments are employed for characterizing crack tips, combining different techniques for a multiscale characterization would benefit our understanding of fracture properties. The advances in fast detector technology and efficient automated analysis routines, shown in all three different fields, allow to combine advanced strain and orientation mapping techniques with *in situ* experiments. This has opened up new avenues for revealing conditions near the crack tips and quantifying crack-tip driving forces. It is expected that local quantitative measurements will not only enable a more direct comparison between experiment and simulation, but also allow to tackle materials containing nanoscale heterogeneities. Understanding the evolution of the stress field during crack propagation at the scale of individual defects is expected to give important input for the design of advanced materials.
